# Brain sodium MRI-derived priors support the estimation of epileptogenic zones using personalized model-based methods in epilepsy

**DOI:** 10.1162/netn_a_00371

**Published:** 2024-10-01

**Authors:** Mikhael Azilinon, Huifang E. Wang, Julia Makhalova, Wafaa Zaaraoui, Jean-Philippe Ranjeva, Fabrice Bartolomei, Maxime Guye, Viktor Jirsa

**Affiliations:** Aix Marseille Université, INSERM, Institut de Neurosciences des Systèmes (INS) UMR 1106, Marseille, France; Aix Marseille University, CNRS, CRMBM, Marseille, France; APHM, Timone University Hospital, CEMEREM, Marseille, France; APHM, Epileptology and Clinical Neurophysiology Department, Timone Hospital, Marseille, France

**Keywords:** Sodium MRI, Bayesian inference, Personalized brain network models, Logistic regression, Imbalanced dataset, Drug-resistant focal epilepsy

## Abstract

Patients presenting with drug-resistant epilepsy are eligible for surgery aiming to remove the regions involved in the production of seizure activities, the so-called epileptogenic zone network (EZN). Thus the accurate estimation of the EZN is crucial. Data-driven, personalized virtual brain models derived from patient-specific anatomical and functional data are used in Virtual Epileptic Patient (VEP) to estimate the EZN via optimization methods from Bayesian inference. The Bayesian inference approach used in previous VEP integrates priors, based on the features of stereotactic-electroencephalography (SEEG) seizures’ recordings. Here, we propose new priors, based on quantitative ^23^Na-MRI. The ^23^Na-MRI data were acquired at 7T and provided several features characterizing the sodium signal decay. The hypothesis is that the sodium features are biomarkers of neuronal excitability related to the EZN and will add additional information to VEP estimation. In this paper, we first proposed the mapping from ^23^Na-MRI features to predict the EZN via a machine learning approach. Then, we exploited these predictions as priors in the VEP pipeline. The statistical results demonstrated that compared with the results from current VEP, the result from VEP based on ^23^Na-MRI prior has better balanced accuracy, and the similar weighted harmonic mean of the precision and recall.

## Author Summary

For the first time quantitative ^23^Na-MRI were used as prior information to improve estimation of epileptogenic network (EZN) using VEP pipeline, a personalized whole-brain network modeling from patient’s specific data. The prior information of EZN can be derived from ^23^Na-MRI features using logistic regression predictions. The ^23^Na-MRI priors inferred EZNs has a better balanced accuracy than the previously used priors or the no-prior condition.

## INTRODUCTION

Epilepsy is a neurological disorder that affects about 1% of the world population, of which approximately 30% are drug-resistant (Picot, Baldy-Moulinier, Daurès, Dujols, & Crespel, 2008). The epileptogenic zone (EZ), corresponding to the cerebral region generating the seizure, might be arduous to locate, and its localization is crucial in refractory epilepsy that requires surgery. Indeed, surgery success is based on the accurate delineation of the EZ, but this area is rarely reduced to a limited brain region (Bartolomei, Wendling, Bellanger, Régis, & Chauvel, 2001), hence the name of “epileptogenic zone network” (EZN) (Bartolomei et al., 2017) used in the following. Great efforts are being made to find objective and quantifiable markers of EZN including interictal markers such as spikes and high-frequency oscillations (HFO), or ictal markers such as the Epileptogenicity index (EI) (Bartolomei, Chauvel, & Wendling, 2008; Scholly et al., 2019). Therefore SEEG recordings are still the gold standard to define EZN, and allows identification of propagation networks (PZ) as well as regions noninvolved by electrical abnormalities (NIZ).

The virtual epileptic patient (VEP) is the personalized whole-brain model for the estimation of EZN using patient-specific data (Jirsa et al., 2023; Jirsa et al., 2017; Wang et al., 2023). The VEP contains modules providing an estimation of the EZN, but also modules virtualizing surgery strategies (Wang et al., 2023). The structural scaffold of the patient-specific whole-brain model is constructed from anatomical T1 weighted MRI, and the network from diffusion-weighted MRI. Each network node is equipped with a mathematical dynamical model, called Epileptor (Jirsa, Stacey, Quilichini, Ivanov, & Bernard, 2014), to simulate seizure activity. Bayesian inference methods sample and optimize key parameters of the personalized model using functional stereo-EEG recordings of patients’ seizures (Jha, Hashemi, Vattikonda, Wang, & Jirsa, 2022; Vattikonda et al., 2021). These key parameters together with their personalized model determine a given patient’s EZN. The VEP provides the fully nonlinear system analysis of whole-brain neural mass models and works on the whole-brain source spaces rather than the sensor recording spaces alone. Epileptor is a phenomenological model based on a system of coupled nonlinear differential equations with five state variables. All together, these equations generate epileptic dynamics called seizure-like events (SLEs). The parameter *x*_0_ in Epileptor, the excitability for each brain region, is a key parameter to lead the system switch between the normal and ictal states (Houssaini, Ivanov, Bernard, & Jirsa, 2015). The VEP model inversion has been proposed to estimate the parameters in order to best fit SEEG recordings data. In addition, the VEP has been compared in a retrospective study of 53 patients to EI type quantification methods and to clinical analysis showing encouraging performances (Makhalova et al., 2022; Wang et al., 2023). The intrinsic nonlinear dynamics of neural mass models in addition to a large number of model parameters and observations render this inversion problem challenging. To solve this problem in Bayesian inference framework (Aster, Borchers, & Thurber, 2018; Gelman et al., 2013), the usage of priors is paramount since it ensures efficient exploration of the posterior distribution by constraining the parameter space (Hashemi et al., 2021). Several prior knowledge can be incorporated such as plausible range of model parameters, dynamics of unobserved brain state, MRI lesions or even the clinical hypothesis of EZN, for instance. The previous VEP priors were mainly based on delay information from filtered SEEG signals in multiple frequency bands during seizure onset or directly from clinical hypothesis (Makhalova et al., 2022; Wang et al., 2023). It is important to explore other neuroimaging modalities as an additional knowledge and as the prior to complement and potentially improve the identification of EZN in the VEP. Moreover, to avoid invasive recordings such as SEEG in the future, an important objective is to feed it with noninvasive data. Here, we explored the potential contribution of ^23^Na-MRI, complementing a recent study seeking to evaluate the link between ^23^Na-MRI measures and excitability, that is, *x*_0_, to eventually demonstrate how such imaging techniques can complement the in silico diagnosis of the EZN.

^23^Na-MRI is the only way to noninvasively quantify sodium in the brain in vivo. However, it can be challenging as the sodium signal is weak compared to proton signal (Madelin, Lee, Regatte, & Jerschow, 2014). In epilepsy, the first ^23^Na-MRI study performed at 3T in a group of human focal epilepsy showed a significant increase of total sodium concentration (TSC) in EZN compared to propagation zone (PZ) and noninvolved zone (NIZ) (Ridley et al., 2017). Nevertheless, TSC has limited specificity for epileptogenicity as it likely reflects intracellular and/or extracellular changes as well as differences in cell density or organization. The quadrupolar interactions of the 3/2 spin of sodium with the electric field gradient of surrounding molecules (Rooney & Springer, 1991) dictate variation in *T*_2_* decay behavior, of which a multiparametric investigation has been made with the biexponential fit of the *T*_2_* decay of the sodium MR signal (Ridley et al., 2018). In this article, we used ^23^Na-MRI at 7T with the enhanced signal-to-noise. The study of quadrupolar interactions gives an indication of the tissue organization and the molecular environment. Bi-exponential of the *T*_2_* decay enables the characterization of the apparent short fraction sodium concentration (*Na*_*SF*_) and the apparent long fraction (*Na*_*LF*_), which when added together gives the TSC (Ridley et al., 2018). In addition, by quantifying the sodium signal fraction with the short *T*_2_* decay component (*f*) this approach may offer a more relevant metric for studying tissue alterations and potentially provide a better link between sodium homeostasis and neuronal excitability in human epilepsy. In a recent study, an increase of *f* in the EZN compared to controls and to PZ and NIZ has been reported, whereas TSC was increased in all regions, including PZ and NIZ (Azilinon et al., 2023).

We hypothesized that ^23^Na-MRI data can provide complementary knowledge to the VEP through prior. Thus in this paper, we aimed to investigate the predictive power of the combination of ^23^Na-MRI features for identifying EZNs. To do so, we explored whether or not the priors derived from ^23^Na-MRI can be used in the VEP framework and evaluated their efficiency. In order to study the individual patterns and to find common features between the different patterns, we combined the different sodium features via machine learning approaches using classification models to predict the possible EZN. We then exploited the sodium features derived EZN candidate as priors in the VEP framework and compared their efficiency to the current used VEP in the clinical trial, Epinov.

## RESULTS

### VEP Workflow With ^23^Na-MRI Prior

**Figure 1.**
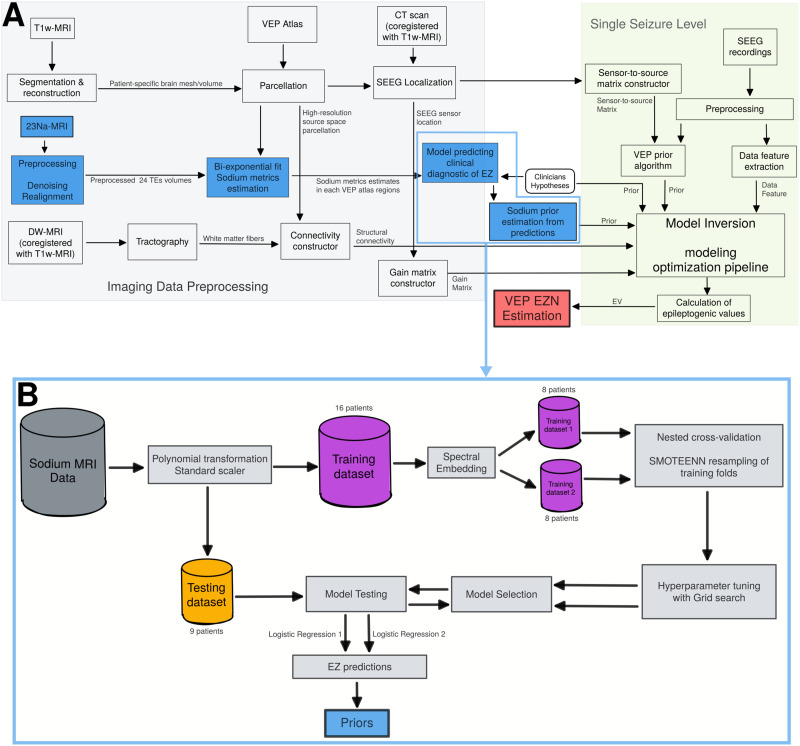
The whole workflow, including flowchart of the VEP pipeline and flowchart of the estimation of the ^23^Na-MRI based priors. (A) In the VEP pipeline, the T1-weighted images define the patient-specific high-resolution space and are used for the parcellation (brain regions on each vertex) according to the VEP atlas. Co-registration of diffusion-weighted images and CT onto T1-weighted images gives structural connectivity, gain matrix, and sensor-to-source matrix in the patient specific brain space. VEP pipeline is run for each seizure, providing EV distribution as well as the diagnosis, and the goodness of fit for the MAP algorithm. (B) ^23^Na-MRI volumes at the 24 echo times (TE) are preprocessed before the biexponential fit estimating the ^23^Na-MRI features extracted into VEP atlas ROIs. The quantitative data are transformed and scaled. The training dataset is splitted into two subsets according to the spectral embedding first eigenvector values as described in the Methods section. Both training datasets were resampled for providing more data points. Hyperparameters were tuned to refine the model selection procedure. Selected models are tested on the testing dataset and the predictions are used as priors.

The VEP workflow, in Figure 1, starts from clinical imaging (anatomical and diffusion MRI) and SEEG data to estimate the EZN via a whole-brain network modeling. Briefly, the brain network model is formed by nodes defined by the regions of the VEP atlas (Wang et al., 2021) linked by the structural connectivity, obtained from the patient-specific imaging data. Note that here the network is patient specific. Epileptor, a phenomenological neural mass model, is then used to simulate seizure-like activity on each brain region. The signals are generated in the source space and then projected onto the sensors, thus obtaining the simulated activity on each channel of the SEEG electrodes.

The model inversion module infers the free parameters of the model related to the excitability of EZNs from SEEG data recording. The data features are extracted through SEEG recordings. Here we used the optimization method using the L-BFGS algorithm, the goal is to obtain the maximum of the posterior distribution of the model parameters, called maximum a posteriori (MAP). To do this, 100 MAP estimates are obtained on datasets with a random sensor removed, resulting in a distribution of epileptogenicity values (EVs) for each region. The EV is calculated by considering the seizure delay in the source level activity based on optimized parameters including excitability of each brain region and structural connectivity. The random sensor removed is for robustness of different electrode combinations. The regions are labeled as EZN if the median of its EVs distribution > 0.6 and confidence > 75%.

Data feature is the power envelope of the signal extracted from the SEEG recordings. We used priors based on ^23^Na-MRI data. We first identified the combinations of ^23^Na-MRI features via a machine learning model called logistic regression to predict the EZN prior. We used the clinical hypotheses as targets in the classification model to predict these hypotheses with the ^23^Na-MRI features. The predictions of the two models tested were used as priors (Na-MRI priors 1 and 2).

### EZN Estimation: Clinical Use Case

The VEP workflow was applied to recordings and imaging data from a 17-year-old female patient, with no surgical intervention yet. The patient was initially diagnosed with bilateral temporal plus epilepsy and a radiologically observed bilateral periventricular nodular heterotopia (Supporting Information Table S1).

The structural connectivity matrix (Supporting Information Figure S1) and sensor-to-source mapping (Supporting Information Figure S2) were extracted from patient T1-weighted image, diffusion weighted images, and postimplantation CT scan data. These matrices, alongside the data features of SEEG seizures recording, were used as input to run the optimization pipeline. Supporting Information Figure S3B exhibits the distribution of the signal power among all electrodes, alongside one recorded seizure (Supporting Information Figure S3A). We can observe here a high activity in the left and right anterior hippocampi in the studied seizure. The VEP prior algorithm takes into account the onset delay of the seizure in each channel while computing sensor prior vector based on 52 different frequency bands from 10–110 Hz. This prior vector is then projected at source level (i.e., brain regions) in two different ways, providing two distinct priors: VEP-M (Supporting Information Figure S4) directly maps the prior value of the sensor with the shortest distance to a given source, while VEP-W (Supporting Information Figure S5) maps a weighted sum of the prior values of all sensors (i.e., all SEEG electrodes) to each source, based on their distance (see Methods).

**Figure 2.**
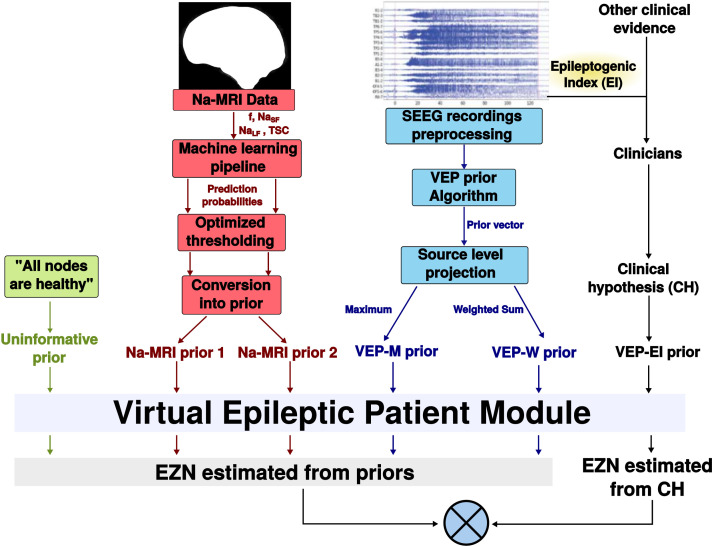
Diagram of comparison of VEP based on different defined prior. We compared the results of VEP based on five different priors: uninformative prior where all nodes are assumed healthy; two Na-MRI prior from Na-MRI data features; and VEP-M and VEP-W prior from SEEG recordings. The compared golden standard is the results of VEP based on clinical hypothesis. These procedures are detailed in section Estimation of Prior and ^23^Na-MRI-based EZN Prediction as Prior.

We also get two other priors from ^23^Na-MRI features. We performed a classification of the regions investigated by clinicians using SEEG, that is, predictions were made on the set of VEP atlas regions that were investigated with SEEG and that were included in the EI analysis. Using the ^23^Na-MRI features (*f*, *Na*_*SF*_, *Na*_*LF*_, and *TSC*) we first trained classification models as described in Figure 1B in order to predict the clinical hypothesis of EZN, as we do not have access to ‘gold standard’ surgery outcome for all the patients included in this prospective study. Features were extracted in the considered regions of interest (ROIs), transformed, rescaled, and the most important were selected as classification models predictors (see Methods). The training dataset is composed of patients that would not be virtualized in the present study, as only patients from the testing dataset would be. Logistic regression was tuned using Grid search function, and after model selection procedure, two logistic regressions (with different hyperparameters and different features) were selected. The predictions made on the testing dataset were used as two distinct priors: Na-MRI prior 1 and Na-MRI prior 2. Hence, we ran the optimization pipeline on six different prior-based networks listed in Figure 2: Na-MRI prior 1, Na-MRI prior 2, VEP-M, VEP-W, uninformative prior, and clinical hypothesis.

**Figure 3.**
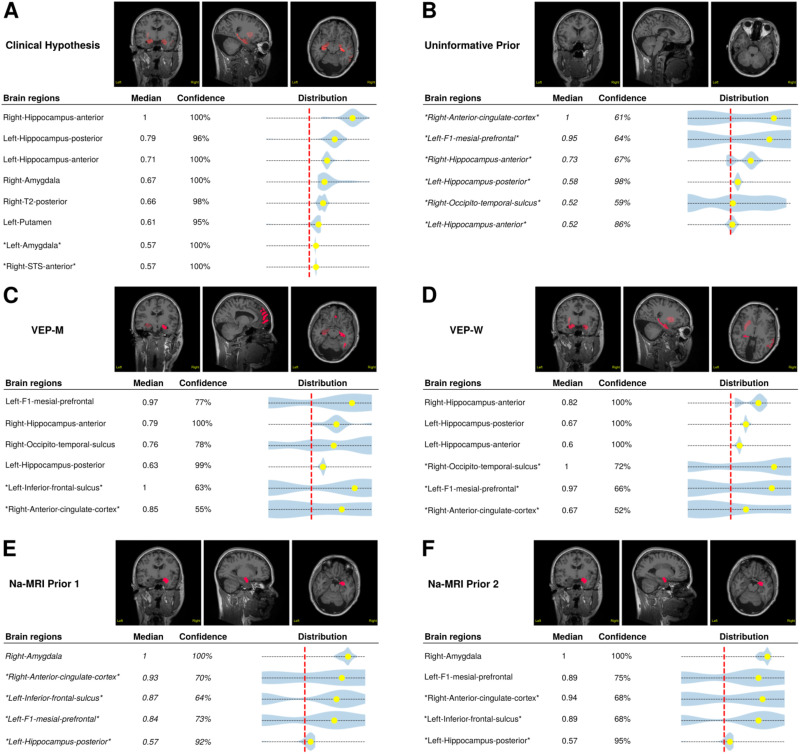
The results of six VEP pipeline based on different priors for the example patient. The results of the VEP based on the prior using (A) the clinical hypothesis; (B) uninformative prior; (C) the VEP-M prior; (D) the VEP-W prior; (E) the Na-MRI prior 1; and (F) the Na-MRI prior 2. Inside each subfigure, on top, the VEP results are labeled in MRI slides, and on bottom, the clinical report tables, where brain regions are selected when median > 0.5 (red dotted line) and confidence > 75%.

Neurologists (JS and FB) furnished the clinical hypothesis based on the EI index (Bartolomei et al., 2008) and other SEEG data, such as direct electrical stimulations. In this clinical use case, we showed that ^23^Na-MRI priors complement VEP priors, particularly VEP-W, which together matched the clinical hypothesis. In fact, we were able to retrieve four over six EZNs (the most evident ones) of the clinical hypothesis, three with VEP-W including right and left anterior hippocampi and left posterior hippocampus (Figure 3D) and one, right amygdala with ^23^Na-MRI prior (Figure 3E and F). This example demonstrates that ^23^Na-MRI is a useful tool to help and complement the diagnosis of the VEP framework.

### Feature Importance and Models Tuning and Selection

The permutation feature shows importance benefits from being model agnostic and can be calculated many times with different permutations of the feature. Here we estimated the cross-validated permutation importance, with 10 repeats and with balanced accuracy as scoring metric, over 17 features. The features and the results of this procedure are listed in the Supporting Information Figure S6. Balanced accuracy is the accuracy adapted to imbalanced data and is defined as the arithmetic mean of sensitivity (true positive rate) and specificity (true negative rate). While thresholding at 0.05, we obtained six features for the training dataset 1 and eight features for the training dataset 2. The two sets of features have three common features: *Na*_*LF*_, *TSC*, and *f*^2^. Therefore, we can consider these three features as “universal” epileptogenic markers, as they are important independently of the training dataset. The difference in features highlights the fact that each training dataset has a different pattern selective of EZN. Some features, were totally useless, with a poor permutation feature importance score, such as the categorical features “lesional patient” and “lesional zone,” make this information not meaningful to predict EZN with these models.

The selected features are used in the models for the hyper-parameters tuning stage. The model selection was based on a cross-validated validation score higher than 0.65 and a difference score lower than 0.1, resulting in 12 models selected. Next, models were trained on their respective training dataset and provide a (optimized) prediction for each patient. The model with the highest mean testing balanced accuracy, for each training split, was selected for the next stage. The retained parameters for these two models are summarized in Table 1. Both models get a tolerance C = 10, a L2 regularization, and similar class weight. On the other hand, solvers differ, logistic regression 1 getting a L-BFGS solver, and logistic regression 2 the SAGA solver.

**Table 1.** Table of tuned parameters of each selected logistic regression resulting from the grid search procedure

**Models**	**Solver**	**Class weight**	**C**
***Logistic Regression 1***	L-BFGS	EZN: 3—rest: 0.6	10
***Logistic Regression 2***	SAGA	EZN: 4—rest: 0.57	10

### Model Testing and Probabilities

**Figure 4.**
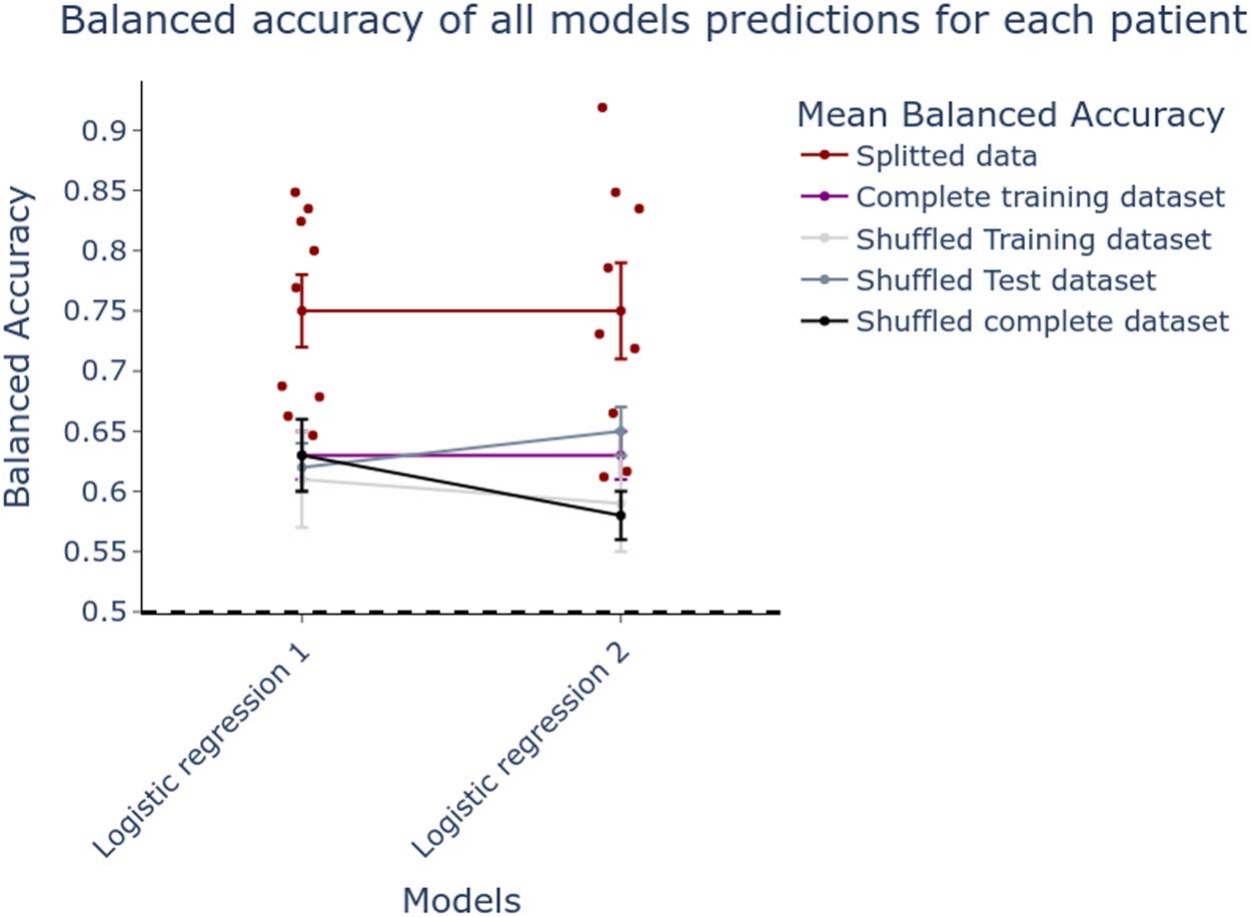
Balanced accuracy of two selected logistic regression prediction results based on ^23^Na-MRI data features against clinical hypothesis. Two logistic regression models were tuned using ^23^Na-MRI data features from two different training datasets, each with eight different patients. We used ^23^Na-MRI data features from nine patients in the test dataset through two trained models (see Table 1) to output their EZNs prediction. Then we calculated the balanced accuracy by comparing obtained EZNs with clinical hypothesis (shown in red dots). Dot plus-minus error bars represent the mean balanced accuracy plus-minus standard error of the mean. When the two models are trained on the complete training dataset (all 16 patients without splitting), the mean balanced accuracy is in purple. Shuffled means that the clinical hypotheses based target labels (i.e., classes to predict) were randomly shuffled. Here we estimate the mean balanced accuracy while shuffling only the training dataset (light gray), only the testing dataset (dark gray), and the whole dataset (black).

Before converting prediction into *x*_0_ and running simulations in the VEP framework, we evaluated performances of the two best logistic regressions, tuned using each training subdataset. Model performances were evaluated against clinician hypothesis about the EZN. Due to variability of ^23^Na-MRI data features between patients in epilepsy, we explored whether a training dataset split can give a more homogeneous training subdataset, and thus better model performance. Thus, the training dataset was split into two subsets according to the spectral embedding first eigenvector values as described in the Methods section. We found out that logistic regression predictions are better on average when using split training dataset compared to the three surrogate models with shuffled targets and to models fitted on the whole training dataset without any splitting. This shows that our splitting approach using spectral embedding was quite efficient. The performances obtained on models trained with the whole dataset with the correct labels are comparable to those of the surrogate models (Figure 4).

The predictions used to estimate the balanced accuracy were binarized model probability predictions. The threshold was optimized for each model and each patient in order to obtain the best prediction of the EZN. These probabilities are represented in Supporting Information Figure S7 for each region’s clinical hypothesis. We observe a clear gradient, with higher probabilities in effective EZN—in the clinical hypothesis—and lower probabilities in NIZ, while PZ probabilities are in between. It is likely that this is due to the ^23^Na-MRI features used as predictors.

### Comparison of Different Priors

**Figure 5.**
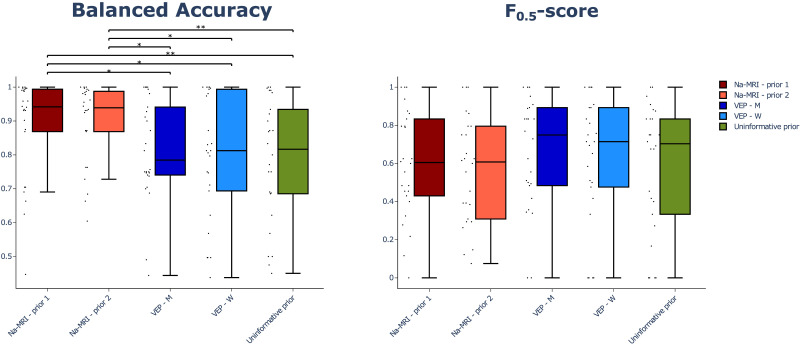
Performance evaluation results. Balanced accuracy (left) and *F*_0.5_-score (right) for each prior EZN estimates for each patient’s seizures against VEP-EI prior EZN estimates. Black dots represent score value for a given seizure on a given patient. Scores of ^23^Na-MRI based priors are represented in shades of red, and those of the currently implemented priors in the VEP (VEP-M and VEP-W) are in shades of blue. Scores of uninformative prior are illustrated with the green box. The top and the bottom of the rectangle in the boxplot represent the first and the third quartile, the line representing the median, and the error bars the extrema. *p < 0.05. **p < 0.01.

At the group level, we analyzed 26 seizures from nine patients as the test dataset using the VEP pipeline. We used five priors for the model inversion on each seizure: Na-MRI-prior 1, Na-MRI-prior 2, VEP-M, VEP-W, and VEP-no-prior. Na-MRI-prior 1 and Na-MRI-prior 2 are derived from predictions of the previous section logistic regression 1 and logistic regression 2, respectively. Parameters resulting from model inversion permit simulation proper to each prior. This was also done for the clinical hypothesis of the EZN, using the VEP-EI prior, and the resulting EZN estimation was used as reference in the performance evaluation approach. Evaluation of performance was made with two different metrics specifically used while dealing with imbalanced data: balanced accuracy and *F*_0.5_-score (Figure 5). These scores were computed for each prior’ EZN estimation against the EZN estimation of the VEP-EI prior. Bootstrapped paired *t* test shows a significantly (*p* < 0.01) higher accuracy of Na-MRI-prior 1 and Na-MRI-prior 2 compared to no-prior. We also can visually see the higher balanced accuracy compared to VEP-M and VEP-W priors, but with lower significance (*p* < 0.05). Bootstrapped paired *t* test does not identify any significant difference of *F*_0.5_-score between priors. By comparing the EZN defined by the ^23^Na-MRI feature against clinical hypothesis shown in Figure 5, the results from VEP with the ^23^Na-MRI feature have higher balanced accuracy.

## DISCUSSION

For the first time we used quantitative ^23^Na-MRI as additional knowledge to help estimation of EZN using the model-based method of VEP pipeline. The priors were based on logistic regression predictions of the EZN, using ^23^Na-MRI features as predictors. The classification procedure outcomes confirmed the existence of variability in sodium feature patterns relative to epileptogenicity, since the use of two training subsamples proved to be more efficient than the use of the whole sample. The procedure provided two models (corresponding to each subsample) built from parameters and features selected specifically for each of them. The predictions of the two models reached an average balanced accuracy of 0.75, much better than those of the surrogate models or the models trained on the whole training set. The ^23^Na-MRI priors inferred EZNs significantly closer to the clinical hypotheses than the currently used priors or the no-prior, in terms of balanced accuracy, but not for *F*_0.5_-score. No significant difference in *F*_0.5_-score reflects no significant difference in precision, as *F*_0.5_-score is rather weighted with precision (positive predictive value). On the other hand, balanced accuracy of the prediction, and therefore the sensitivity (true positive rate) and specificity (true negative rate) with each other are significantly improved while using ^23^Na-MRI priors.

### ^23^Na-MRI Features

The feature engineering methodology makes it impossible to evaluate the contribution of each ^23^Na-MRI feature. Most ^23^Na-MRI studies (the vast majority at 3T) have shown an increase in TSC in neurological diseases such as ALS (Grapperon et al., 2019), Huntington’s (Reetz et al., 2012), Parkinson’s (Grimaldi et al., 2021), multiple sclerosis (Maarouf et al., 2017; Zaaraoui et al., 2012), and, of course, epilepsy (Ridley et al., 2017). Using 7T MRI we also used *f*, *Na*_*SF*_, and *Na*_*LF*_, measures estimated from the biexponential fit parameters of the 24 TEs (see section Data Processing). *f* reflects the apparent ratio of short and long *T*_2_* sodium signal decays, and thus encompasses the smallest measurable effects at each TE, with a weighting for short TEs. While in free liquid such as the CSF, the *T*_2_* signal decay is mono-exponential, the quadrupolar interaction of sodium nuclei with the electric field of molecules lead to bi-exponential *T*_2_* decay in the tissues (Berendsen & Edzes, 1973). Here we assumed that *Na*_*SF*_ and *Na*_*LF*_ will be important in the characterization of EZN via logistic regression; in fact, both measurements together constitute TSC, hence we have the opportunity to investigate whether or not they can refine the characterization of EZN when not encompassed in TSC, which appear to be crucial for the prediction of the EZN (Supporting Information Figure S6). We could therefore imagine in the future to refine these measures by improving the compartmental models of sodium (multiexponential decays accounting for intracellular, extracellular, CSF, and vascular compartments) for a better characterization of the epileptogenic network.

Together, these measurements provide information on several aspects: (i) sodium homeostasis; (ii) microstructure, as the sodium signal may also reflect the structure of the medium in which the sodium is located (Rooney & Springer, 1991); and (iii) variation in perfusion (already demonstrated in epilepsy (Kojan et al., 2021; Wang et al., 2018)), which may impact blood sodium signal as well, not negligible in the total sodium signal (Driver, Stobbe, Wise, & Beaulieu, 2020). It should also be kept in mind that unlike SEEG, which records electrical activity emanating from neurons, ^23^Na-MRI measures the above-mentioned phenomena in the tissue and thus in neurons and glial cells. Thus, when the SEEG is clearly designated as measuring excitability, we are able to ask ourselves, in view of the results, whether the combination directly measures cortical excitability. We can also ask whether these metrics can be used to analyze phenomena involved in the ongoing epileptogenesis, such as inflammation, glial reaction, plasticity, or reorganization (Scharfman, 2007).

### Predictive Model

Being in a supervised training paradigm, we studied the different feature patterns within the EZN in the training dataset. The complete training dataset gave poor performance. Indeed, the raw data showed various patterns, specific to each patient. To provide consistent datasets, we used Spectral Embedding for feature reduction, and we used the eigenvector resultant to split the data into two subsamples. In the context of epilepsy, this is easily explained because in our sample, epilepsies are heterogeneous in terms of causes, disease duration, and location and anatomical organization. These differences should involve various ionic and metabolic changes in the tissue. Presumably, the patterns of sodium features should vary with these differences. This aspect could not be addressed in this study because of the small number of patients with each type of epilepsy. In the future it may be interesting to explore this issue.

Polynomials and interactions allow the models to learn a more complex decision boundary, thanks to the nonlinearity introduced (Kuhn & Johnson, 2019). However, overfitting becomes a risk and interpreting feature importances gets trickier. In fact, it is difficult to biologically interpret feature combinations because the permutation value of a feature combination is relative to other combinations. All these weaknesses might be addressed in future works. In this work we rather aimed to define an accurate and efficient marker of epileptogenicity than an interpretable model. Nevertheless, in the case of two correlated features, if one of the features is permuted, the model will still have access to the feature through its correlated feature. This will induce a lower importance value for both features, while they may be important. Here, there are indeed correlated features, which expose us to this bias. But we have other downstream strategies to manage the possible biases induced, like the L2 regularization of the models, which will minimize the impact of the ‘useless’ features. In case we missed a feature, we can live with that as long as the model classifies well.

Studying logistic regression predictions probabilities and searching for the optimal threshold for the best performance (balanced accuracy score), we observed that the model seems to be sensitive to clinical hypotheses. In fact, there is a gradient of prediction probabilities with EZN the higher, NIZ the lower, and PZ in between. This means that although the model was trained on binary targets (EZN vs. non-EZN, i.e., PZ and NIZ), the ^23^Na-MRI features used as predictors show that PZs are not quite NIZs nor quite EZNs. The average probability of EZN is very high (around 0.8), that of NIZ is below 0.4, while that of PZ is slightly higher than NIZ. This is even more pronounced in logistic regression 2 than in logistic regression 1 (Supporting Information Figure S7) showing that the model is rather indecisive about PZ. It would be very interesting to study multiclass classification in this context, which would also address the issue of regional variance.

### VEP Pipeline and Model Inversion

The clinical hypothesis used here depicts the epileptogenic zone network (EZN), the propagation zone (PZ) and the noninvolved zone (NIZ), but to simplify the procedure we have binarized these assumptions—corresponding to our classification targets—considering a “one vs the rest” strategy, since we are interested in the EZN specifically. This is a strong assumption that affects the choice of *x*_0_ (either −1.5 for a strong excitability or −3 otherwise). Nevertheless, when we look at the probabilities of the models, we notice a gradient, which shows that the models consider the PZs as fuzzy zones between pathological and healthy excitability. It also reflects a continuum of the excitability, referred to in the literature (Bartolomei et al., 2008) We could therefore deepen this study by making a multiclass classification aiming at predicting the EZN, the PZ, and the NIZ, to then fix the values of *x*_0_ according to each prediction, with an *x*_0_ between −1.5 and −3 for the propagation zones.

Here we use MAP for the VEP model inversion. The main advantage of MAP is that it is not computationally expensive. However, MAP can get stuck in a local extrema as other optimization algorithms. One of the solutions is to use MCMC-based sampling methods such as NUTS or HMC (Betancourt, 2017; Hoffman & Gelman, 2014). It was recently shown that HMC sampling implemented in the VEP provides similar results. But in the future, to confirm the results of the present study, or to refine the estimation of EZN, more robust (but also more expensive computationally) techniques like HMC sampling could be employed.

The parameters provided by the optimization procedure using MAP tune the neural mass model, which in turn generate simulated brain activity of a bulk group of neurons. Indeed, reducing 1,000 vertices of source activity into one node mapped to a VEP region cancels out the directionality of the current dipole of the folded cortical sheet, which may lead to wrong mapping from sources to sensors and thus possibly introduce errors into the estimation of EZN. The neural field model (NFM) might be a solution, simulating brain activity at the vertex level. But the relatively low resolution of ^23^Na-MRI will complicate the mapping of sodium features onto the vertices. Moreover, the important bias that partial volume effect might introduce in the vertex level sodium estimates will eventually mistake the ^23^Na-MRI based priors. An improvement of the ^23^Na-MRI resolution will facilitate the approach.

Multiple aspects of focal seizures may vary, such as causes, electrographic onset patterns, duration and underlying dynamics. Moreover, it can also happen in a given patient. Our data contains all this possible variability. A priori this variability can be explained by seizure-specific excitabilities (VEP-M and VEP-W) combined with patient-specific structural connectivity. On the other hand, ^23^Na-MRI priors do not take into account the seizure-specific aspect, since only one interictal prior will be used for all seizures of a given patient. Interestingly, this did not have any impact on the outcome, given the high balanced accuracy obtained for ^23^Na-MRI priors. This may mean that commonalities between different seizures could be detected with noninvasive interictal measures such as ^23^Na-MRI, but more studies are needed to make this claim.

In future, the usage of ^23^Na-MRI-based priors could pave the way for the current VEP protocol toward noninvasive diagnosis. In theory, any other quantitative measure, such as positron emission tomography (PET), can be tested and used as prior in our modeling approach. We are also testing the VEP pipeline on noninvasive electrophysiology data, such as MEG and EEG, to predict the EZN noninvasively. These offer ways to transfer new relevant methods to clinical practice.

### Limitations and Perspectives

In the present study, we based data selection on the clinical hypothesis which lies on SEEG recording analysis and implantation spatial sampling, considering it as ground truth. As SEEG recordings analysis may suffer from spatial sampling problems, using it as ground truth is debatable, especially when the ground truth is usually considered to be the brain region which once removed leads to no more seizures (Lüders, Najm, Nair, Widdess-Walsh, & Bingman, 2006). Nevertheless, we had to make a choice for a ground truth using such a prospective database, where the majority of the patients have not had a surgery, and the choice was to use the best estimation of the EZN common to all patients at the moment of this study. In future, applying a similar approach on seizure-free patients only will be needed to confirm these results.

We have considered a normal distribution of excitability to compute parameter likelihood. The excitability of a region, in the context of a phenomenological model such as Epileptor, is the cumulative sum of the effects of the components that play a role in seizure generation. If these components can be random independent variables then, according to the central limit theorem (Sip et al., 2021), their sum converges to a normal distribution. However, we can imagine that in the case of some epileptogenic lesions, which may or may not generate seizures, a bimodal distribution would be more appropriate.

This last point can also be improved by introducing the regional variance. Currently the parameterization is identical for all the parameters of the model except *x*_0_, and this for all the sources. It would be interesting to vary the parameters according to other biological information, such as cell density, cell type within a region, as well as functional specialization of brain regions. The regional variance can also depend on the functional specialization of regions or the structural connectome. Regional activity variance has been demonstrated using power spectra and peak frequency of functional data such as SEEG (Frauscher et al., 2018). Most of the anatomical and functional data related to regional variance are available at the group level, making it challenging to use this information in an individualized approach like the one used in this study. In the future it would be interesting to explore how ^23^Na-MRI data can provide information in this sense; now that we are able to extract ^23^Na-MRI data in the whole brain via VEP atlas, we need to determine the right model parameters which can be tuned based on these data to address regional activity variance from the homeostatic point of view. Virtual brain twins have been extended from VEP to various brain disorders, such as Parkinson’s and multiple sclerosis, among others (Wang et al., 2024). Sodium MRI shows promise in these domains and may offer the useful personalized information that enhances the clinical utility of virtual brain twins (Grimaldi et al., 2021; Maarouf et al., 2017).

## CONCLUSION

For the first time, quantitative ^23^Na-MRI was used in the VEP framework. One of the main results of this study is that ^23^Na-MRI features help the VEP to better predict the EZN in terms of a high balanced accuracy when taking the clinical hypotheses as the ground truth. Combining ^23^Na-MRI features via a machine learning approach appears to be a relevant tool to predict the clinical hypothesis and, therefore, the derived prediction used as priors in the VEP pipeline can provide more information and a new point of view about the EZN. The next steps would be to upgrade this approach, considering multimodal imaging data, combining other quantitative imaging modalities such proton spectroscopy imaging, as input data to the machine learning pipeline as well as surgery-defined EZN as targets, aiming for more precise estimation of the EZN.

## MATERIALS AND METHODS

### Data Acquisition

We obtained the dataset from 25 patients with drug resistant focal epilepsy who underwent a standard presurgical protocol at La Timone hospital in Marseille. Informed written consent was obtained for all patients in compliance with the ethical requirements of the Declaration of Helsinki, and the study protocol was approved by the local Ethics Committee (Comité de Protection des Personnes sud Méditerranée 1).

Patients’ clinical records, neurological examinations, neuropsychological testing, and EEG recordings were assessed in the noninvasive evaluation. The subjects’ clinical data are given in Supporting Information Table S1. The evaluation included noninvasive *T*_1_-weighted imaging (see MRI Acquisition section of the article for more information) and diffusion-weighted images (DTI-MR sequence, either with an angular gradient set of 64 directions, repetition time = 10.7 s, echo time = 95 ms, voxel size 1.95 × 1.95 × 2.0 mm^3^, b-weighting of 1,000 s × mm^2^, or with an angular gradient set of 200 directions, repetition time = 3 s, echo time = 88 ms, voxel size 2.0 × 2.0 × 2.0 mm, b-weighting of 1,800 s/mm^2^) acquired on a Siemens Magnetom Verio 3T MR-scanner.

In addition, ^23^Na-MRI was acquired using a dual-tuned ^23^Na/ ^1^H QED birdcage coil and a multiecho density adapted 3D projection reconstruction pulse sequence on a whole-body 7-Tesla Magnetom Step 2 MR system (Siemens, Erlangen, Germany) (for acquisition information see MRI Acquisition section of the article). To ensure a sufficient number and distribution of TEs, 3D ^23^Na MRI volumes were obtained at 24 echo times (TEs) ranging from 0.2 ms to 70.78 ms. This approach also takes into account the 5-ms readout of the sequence, needed to acquire ^23^Na signal with short *T*_2_* decay. Signal quantitative calibration into sodium concentration was performed using six tubes (80-mm length, 15-mm diameter) filled with a mixture of 2% agar gel and sodium at different concentrations: two tubes at 25 mM, one at 50 mM, two at 75 mM, and one at 100 mM. These external references were positioned in the field of view in front of the subject’s head and maintained using a cap.

Implanted depth electrodes provide patients’ invasive electrophysiological recordings. Electrodes used in SEEG contain 10–18 contacts 2 mm long, which are spaced by 1.5-mm or 5-mm gaps. The SEEG signals were acquired on a 128 channel Deltamed/Natus system with at least a 512 Hz sampling rate and recorded on a hard disk (16 bits/sample) using no digital filter. A high-pass filter (cut-off frequency equal to 0.16Hz at −3dB) was used in the acquisition procedure, as well as an anti-aliasing low-pass filter (cutoff at one third of the respective sampling frequency). SEEG electrodes location are obtained by dint of cranial CT scan, performed after the implantation of electrodes.

### Data Preprocessing

To construct the individual brain network models, we performed a volumetric segmentation and cortical surface reconstruction from the patient-specific *T*_1_-MRI data using the recon-all pipeline of the FreeSurfer software package (https://surfer.nmr.mgh.harvard). The cortical surface was parcellated according to the VEP atlas (Wang et al., 2021) (code available at https://github.com/HuifangWang/VEP_atlas_shared.git).

We used the MRtrix software package to process the DW-MRI (Tournier et al., 2019), using the iterative algorithm described in Tournier, Calamante, and Connelly (2012) to estimate the response functions and subsequently used constrained spherical deconvolution (Tournier, Calamante, & Connelly, 2007) to derive the fiber orientation distribution functions. The iFOD2 algorithm (Tournier, Calamante, & Connelly, 2010) was used to sample 15 million tracts. The streamlines to and from each VEP ROI were assigned and counted, providing the structural connectome. Self-connections, represented by the diagonal of the structural connectome matrix, were excluded by setting them to 0. Finally, the matrix was normalized so that the maximum value was equal to one.

GARDEL (Graphical user interface for Automatic Registration and Depth Electrodes Localization), as part of the EpiTools software package (Villalon et al., 2018), estimated the location of the SEEG contacts from postimplantation CT scans. Afterwards, we coregistered the contact positions from the CT scan space to the *T*_1_-MRI scan space of each patient.

**Figure 6.**
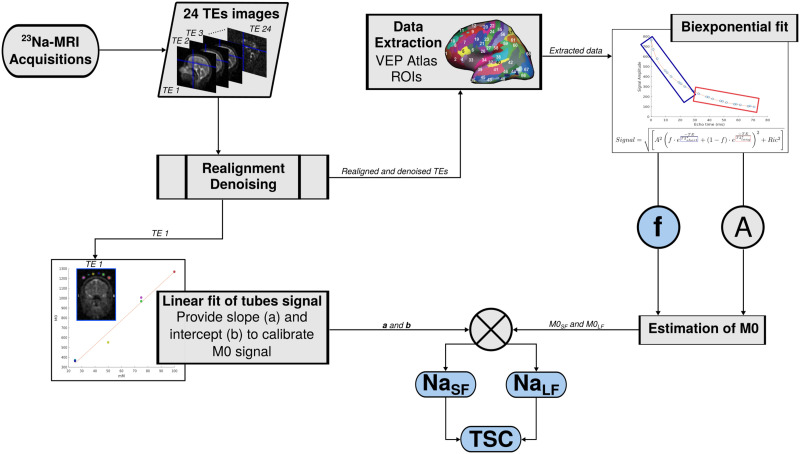
Diagram of ^23^Na-MRI data processing. The 24 volumes acquired with different TEs acquired using a 3D radial density adapted sequence at 7 Tesla are first realigned and denoised. The resulting TE1 image is used for the linear fit of tubes sodium signal to calibrate the sodium signal afterwards. All 24 TE signal intensities are extracted into VEP atlas ROIs and fitted with the biexponential model. The fit provides parameters such as *A* and *f*, that provide *M*0 signals fractions. The resulting short and long *M*0 are calibrated into *Na*_*SF*_ and *Na*_*LF*_. The sum of the resulting *Na*_*SF*_ and *Na*_*LF*_ gives TSC (Azilinon et al., 2023; Grimaldi et al., 2021; Ridley et al., 2017).

^23^Na-MRI data processing is summarized in Figure 6. All 24 TEs acquired were denoised and realigned on the first TE volume in the same fashion as in the article. VEP atlas volume obtained from VEP pipeline data preparation is ‘voxel cleaned’ via cerebrospinal fluid (CSF) mask obtained with SPM segment function from high-resolution *T*_1_ (MP2RAGE) image, after coregistration in the corresponding space. All the voxels shared by this CSF mask and any VEP atlas ROI are erased. This stage was performed in the ultrahigh resolution of the 7T *T*_1_ image space (for more information about the MP2RAGE see MRI Acquisition section of the article). The cleaned atlas was then projected into ^23^Na-MRI native space for extraction of the 24 TE signals into each of the 162 VEP atlas ROIs. The mean signal of each TE in each ROI was then fitted with the biexponential model presented in the first part of this manuscript.

$$\text{ROIsignal}=\sqrt{\left[A^{2}\left(f\cdot e^{\frac{-TE}{{{T_{2}}^{*}}_{\text{short}}}}+\left(1-f\right)\cdot e^{\frac{-TE}{{{T_{2}}^{*}}_{\text{long}}}}\right)+{Ric}^{2}\right]}$$

We derived from the biexponential fitting the magnetization (*M*0) corresponding to the signal fraction estimated by the model in terms of the intercepts of the signal fraction components of the model, obtaining *M*0_*SF*_ = *A* · *f* and *M*0_*LF*_ = *A* · (1 − *f*). Then, *Na*_*SF*_ and *Na*_*LF*_ were calculated with raw *M*0 signal values and the linear fit estimated over the tube phantoms, that is, slope (a) and intercept (b):

$$\begin{array}{lll} {Na}_{SF} & = & \frac{\left(M0_{SF}-a\right)}{b}\\ {Na}_{LF} & = & \frac{\left(M0_{LF}-a\right)}{b}\\ TSC & = & {Na}_{SF}+{Na}_{LF}\\ f & = & \frac{{Na}_{SF}}{TSC} \end{array}$$

### VEP Model Construction

To predict individualized EZNs, whole-brain neural model (Sanz-Leon, Knock, Spiegler, & Jirsa, 2015)—considering brain areas as nodes connected through edges formed with white matter fibers—was used. Basically, the VEP atlas provides the brain regions, the so-called nodes, whereas dWI provides the white matter tracts used to attribute a connection strength to edges. Dynamical equations model the activity of each node, which propagates from one region to another depending on the connection strength between them. The seizure-like activity is modeled with Epileptor, a 6D phenomenological model (Jirsa et al., 2014). The model is composed of three neural populations and three time scales: fast, intermediate, and slow. Taking advantage of time scale separation and using averaging methods, the 6D Epileptor is reduced to a 2D system (Proix, Bartolomei, Chauvel, Bernard, & Jirsa, 2014):

$$\left\{\begin{array}{lll} {\dot{x}}_{i} & = & I_{i}-x_{i}^{3}-2x_{i}^{2}-z_{i}\\ {\dot{z}}_{i} & = & \frac{1}{\tau_{0}}\left(4\left(x_{i}-x_{i,0}\right)-z_{i}+K\sum_{j=1}^{N}C_{i,j}\left(x_{j}-x_{i}\right)\right) \end{array}\right.$$

where *τ*_0_ scales the length of the seizure. The state variable *x*_*i*_ describes the activity of neural populations on a fast time scale and can model fast discharges, mostly during ictal periods. The oscillation of the slow permittivity variable *z*_*i*_ drives the system autonomously between ictal and interictal states. The parameter *x*_0_ indicates the degree of excitability (or epileptogenicity) and directly controls the dynamics of the neural population to produce the seizure or not. The coupling between nodes of the network is defined by *C*_*i*,*j*_, which comes from the structural connectivity. *K* scales the connectivity, which can be varied between simulations to investigate different scenarios. The external input is defined as *I*_1_ = 3.1. In this work we exploited the 2D Epileptor for model inversion in order to speed up the computations essentially.

### Neural Mass Modeling

Neuronal population macroscopic behavior is modeled by neural masses. In TVB (Sanz-Leon et al., 2015), neural masses represent activity of a single brain region, linked through the structural connectivity, forming a complete brain network model. The global equation for such a model can be given by

$${\dot{\psi }}_{i}\left(t\right)=F\left(\psi_{i}\left(t\right)\right)+K\sum_{j=1}^{L}W\left(i,j\right)S\left(\psi_{i}\left(t\right),\psi_{j}\left(t\right)\right)$$

where Ψ_*i*_(*t*) is a state vector of neural activity at brain region *i* and time *t*. $\dot{\Psi }$ is the temporal derivative of the state vector. *F*, a function of the state, captures the local neural activity; *F* reflects the Epileptor model in the present work. *W* is a matrix of heterogeneous connection strengths between nodes *i* and *j*. *S* is a coupling function of the local state Ψ_*i*_ and the distant delayed state Ψ_*j*_. The sum across the number of nodes *L* (scaled by a constant *K*) gives the network input received by a node *i*.

### Forward Solution

Solving the forward problem and estimating a source-to-sensor matrix permits one to map the neural activity from sources (VEP brain regions) to the sensors (SEEG electrods contacts). This matrix *g*_*j*,*k*_ from source brain region *j* to sensor *k*—also called Gain matrix—is equal to the sum of the inverse of the squared Euclidean distance *d*_*i*,*k*_ from vertex *i* to sensor *k*, weighted by the area *a*_*i*_ of the vertex on the surface.

$$g_{j,k}=\sum_{i=0}^{N_{j}}\frac{a_{i}}{{d_{i,k}}^{2}}$$

Region *j* contains the vertex *i*, containing a total of *N*_*j*_ vertices. The area *a*_*i*_ of vertex *i* is obtained by summing up one third of the area of all the neighboring triangles. To obtain the gain for a single region of the brain network model, vertices from the same brain region are summed. The resulting gain matrix has dimensions *M* × *N*, with M being the number of regions and *N* the number of sensors. Matrix product of the simulated activity at the source with the gain matrix gives the simulated SEEG signals.

### Estimation of Prior

Here we provide prior to the model inversion module as *x*_0_ parameter values. Prior was usually estimated from SEEG processing (Hashemi et al., 2020; Wang et al., 2023). The Fourier transform of the SEEG signal provides a spectrogram in 200 consecutive windows. After log transformation of spectrogram values, the seizure onset in each SEEG channel is identified by a sensor-prior vector. The values of the vector are estimated based on channel spectral content across 52 different frequency bands. Each frequency band is defined by the combination of a lower bound ranging from 10 to 90 Hz and an upper bound ranging from lower bound +10 to 120 Hz, both in steps of 10 Hz. Averaging spectrograms across a given frequency band gives an average time series per SEEG channel, which is thresholded by its 90% percentile. Early increase in a specific frequency band is illustrated by high values of normalized reciprocal value (reciprocal of the first time point above the threshold in each channel). This is considered to be a specific indicator of the SEEG channel where seizure begins. These values are mapped to the source brain region, considering the 60 closest vertices to the sensor for each region. The entries of the sensor-to source-matrix corresponds to

$$\frac{1}{60}\sum_{i=1}^{60}\frac{1}{d_{i}^{k}}$$

with $d_{i}^{k}$ being the euclidian distance from sensor *k* to source vertex *i*. The prior VEP-M consists of assigning the sensor-prior value to the region with the strongest projection to a particular sensor in the sensor-to-source-matrix. VEP-W calculates one onset value per region for each frequency band, which is then averaged across all frequency bands and divided by the max. For both strategies, regions with the resulting value higher than 0.5 are assigned with a *x*_0_ = −1.5 (putting a high epileptogenicity on nodes representing those regions), and a *x*_0_ = −3 when lower than 0.5, considering these brain regions as nonepileptogenic. In fact, all brain regions get this *x*_0_ value for VEP-no-prior. For the VEP-EI prior, regions diagnosed as EZN by clinicians are assigned with a *x*_0_ = −1.5 and the other regions with *x*_0_ = −3. Estimation of ^23^Na-MRI-derived priors is detailed in the next section.

### ^23^Na-MRI Based EZN Prediction as Prior

Seeking to predict the clinical diagnosis of EZN with ^23^Na-MRI features, we evaluated the classification performances of logistic regression with ^23^Na-MRI-derived features as predictors. All classification models are binary, where the positive class is “EZN” and the negative class is “not EZN.” Since the data is highly imbalanced (there are much less “EZN” and “not EZN”), a cost-sensitive framework—through the usage of class weights parameter—is necessary to adjust models prediction (Fernández et al., 2018). The “not EZN” label corresponds to the concatenation of PZ and NIZ labels. These labels are mostly diagnosed based on SEEG recording processing, using the Epileptogenic Index (EI) (Bartolomei et al., 2008). Hence, the classification was focused on the region with one of those labels. While setting the procedure, we observe a huge variability in Na features patterns, making the model perform poorly. We then decided to split the training dataset to deal with this issue. We tuned the models on both training datasets. The resulting tuned models were used to predict the EZN in of the test dataset patients, providing two priors for the VEP pipeline. The whole procedure is detailed below. All priors definitions are summarized and illustrated in Figure 2.

### Data Preparation

#### Data split.

The train-test split was performed over the 25 patients dataset, training dataset used only for hyperparameter tuning, model selection and model fitting, and a testing dataset from which we get the predictions of fitted models and use them as priors in the VEP pipeline. Not all of the 25 patients had all the necessary data for the VEP pipeline, so we put those patients into the train dataset. The final training dataset contained 16 patients (9 in the testing dataset).

We observed heterogeneous ^23^Na-MRI feature patterns at the individual level, which initially provided weak performance. So we opted to split the train set to train the models on data with different patterns. We arbitrarily choose to split into two different datasets using spectral embedding (Luxburg, 2007) with 2-dimensional projected subspaces. The patients with the mean value of the first eigenvector in the EZN over 0 were included in the training dataset 1, and the others in the training dataset 2, making two sets of eight patients each. Briefly, spectral embedding is a nonlinear dimensionality reduction using Laplacian eigenmaps, which preserves the local geometry. Here, we used a graph of nearest neighbors to construct the affinity matrix. After this stage, Laplacian decomposition is applied to the corresponding graph Laplacian. The eigenvectors for each data point provide the resulting transformation. Here, spectral embedding dimensionality reduction that preserves the local geometry is crucial to categorize patients with similar ^23^Na-MRI feature patterns. Figure 4 illustrate the added value of this approach.

#### Feature analysis procedure.

The first step consists in applying a polynomial transform to features (degree = 2, with interaction terms), basically creating new input features from existing features. The resulting polynomial features correspond to the initial feature plus squared features and interaction terms of each pair of the initial features, obtaining 15 features, in addition to the two categorical features (17 altogether) considered here: “lesional patient,” binary vector containing 1 for regions of a patient with a lesion, and “lesional zone,” binary vector containing 1 for lesional regions. Thanks to the exponent, this transformation separates small and big values and thus changes the probability distribution. The addition of polynomial features to model inputs allowed the model to identify nonlinear patterns alongside linear patterns (Kuhn & Johnson, 2019). Polynomial transformation was followed by a data standardization step, needed for the majority of machine learning models.

The next step was permutation feature importance, which corresponds to the decrease in the model score when a feature values are randomly permuted (Breiman, 2001). This procedure can be applied multiple times on repeated permutation of a feature (repeated 10 times here). For each model and on both training sets, this approach was validated using nested cross validation. For each cross-validation fold, permutation feature importance got a fitted predictive model and training-split of a fold as inputs. Then it computed the reference score of the model on the data, in this instance balanced accuracy. Next, each feature was randomly permuted 10 times, computing at each repetition the permuted score. The importance was finally obtained from the difference between the reference score and the permuted scores.

The nested cross-validation and the resampling technique used in the hyperparameters tuning were also used in this procedure; the cross-validation providing the training and the validation folds, where the training fold was resampled according to the resampling procedure described in the next section. The so-called permutation importance is estimated by computing the difference between baselines core and permuted score. For each model and training set, the decision threshold of the permutation importance was set to 0.05, considering (arbitrarily) that the most important features should induce a score drop of at least 0.05 when permuted.

#### Model selection procedure and priors estimation.

Feature permutation outcomes are used as predictors in the following steps. Models hyper-parameters were tuned using a grid search function, which searches over a specified set of parameters values (Table 1) the best mixture of parameters. Each mixture provides a model that will fit on the training dataset, and then provides a performance score, here balanced accuracy. Cross-validated grid search over a parameter grid optimizes parameters of the models. We used a nested cross-validation (CV) approach (Wainer & Cawley, 2018), resampling the train-split of each CV fold (Batista, Prati, & Monard, 2004). In our nested CV, the outer fold contains two patients and the inner fold is composed of one patient. For each fold the minority class is oversampled to 60 points while the majority class is oversampled to 180 points before undersampling by ENN, which preserves a relative imbalance, managed by model class weights. The decision about the sets of parameters to use is based on their respective models performance, using the mean cross-validated validation score and the scores difference (mean CV train score − mean CV validation score). The models should have a mean cross-validated score above 0.65 to remove all the models with a performance close to the chance-level. A score difference lower than 0.1 removes all models that overfit, that is, train score is much higher than the validation score.

The parameter combinations retained provide 12 models. In order to reduce the number of models, we have chosen a model for each training dataset—as all models reached similar performance—considering the mean test balanced accuracy maximum value. The parameters and scores of the selected models are listed in Table 1.

Model validation consists in comparing model performance on real data compared with surrogate data (shuffled targets, shuffled training dataset targets, and shuffled testing dataset targets). This last stage defines the models used for the prediction/priors estimation stage. Predictions are made from prediction probabilities defined by each model. These probabilities are threshold in order to optimize the test score and obtain the best prediction possible from those models. The thresholding binarized the probabilities, in order to be integrated into the VEP framework, and more specifically into the Epileptor model. The predictions are then converted into *x*_0_, the neural mass excitability parameter.

### Optimization MAP Pipeline

To infer the epileptogenicity parameter and source time series for each seizure, we apply a Bayesian modeling approach. According to Bayes’ theorem, the posterior probability distribution *p*(*θ*|*y*) of a parameter *θ* given the data *y* is equal to the product of the likelihood *L*(*y*|*θ*) of the data given the parameter and the prior probability distribution *p*(*θ*) of the parameter divided by the marginal likelihood *p*(*y*) of the data.

$$p\left(\theta |y\right)=\frac{L\left(y|\theta \right)p\left(\theta \right)}{p\left(y\right)}$$

In such a complex multivariate models as (Epileptor) VEP, the marginal likelihood

$$p\left(y\right)=\int L\left(y|\theta \right)p\left(\theta \right)dy$$

is unsolvable; but as it scales to 1 the integrale across the posterior distribution, one can state the Bayes theorem as

$$p\left(\theta |y\right)\propto up\left(\theta |y\right)=L\left(y|\theta \right)p\left(\theta \right)$$

Where the posterior probability is proportional to the unnormalized posterior probability *up*(*θ*|*y*). Normalized and unnormalized posterior distributions have the same properties (namely the shape, maxima and minima), but they are scaled by their respective constant versions. Markov chain Monte Carlo methods generate samples of the unnormalized posterior distribution, hence the posterior distribution can be approximated and inference can be made about the parameters of interest, with a sufficient amount of samples. The maximum-a-posteriori estimate *θ*_*MAP*_ was computed in the optimization pipeline using the L-BFGS (Nocedal, 1980) quasi-Newton method.

$$\theta_{MAP}\left(y\right)=\arg\;\max\left[up\left(\theta |y\right)\right]$$

The optimization algorithm performs an iterative process to find *θ*_*MAP*_. It starts with an initial assumption of the parameter before moving through parameter space following the direction of the gradient of the probability distribution. The algorithm terminates after either a maximum of 20,000 steps or convergence has been reached. When changes in parameters, gradients, or probability density between steps are below a certain threshold, convergence is detected. In the current work exploiting the VEP model, the product of prior probability of each parameter and the likelihood of the data provide the posterior probability. Stan (Carpenter et al., 2017) transforms probability into log proba, resulting in a log of posterior proba that corresponds to the sum of log likelihood and log prior probability of all parameters. We specified the prior probabilities for the epileptogenicity parameter *x*_*i*,0_ for brain region *i*, the time scale of the slow variable *τ*_0_, one scaling s and one additive constant of the simulated SEEG, the global coupling scaling factor *K*, and the initial conditions for state variables *x*_*i*_(*t*_0_) and *z*_*i*_(*t*_0_) in region *i*, as well as the distribution width of the extracted data features *ϵ*_*ν*_:

$$x_{i,0}∼𝒩\left(x_{i,m},1\right)$$

$$\tau_{0}∼𝒩\left(20,10\right)\;\text{with}\;5\leq \tau_{0}\leq \infty$$

$$s∼𝒩\left(1,10\right)\;\text{with}\;0\leq s\leq \infty$$

$$a∼𝒩\left(0,10\right)$$

$$K∼𝒩\left(1,10\right)\;\text{with}\;0\leq K\leq \infty$$

$$x_{i}\left(t_{0}\right)∼𝒩\left(-2,10\right)$$

$$z_{i}\left(t_{0}\right)∼𝒩\left(3.5,10\right)$$

$$\epsilon_{\nu }∼𝒩\left(1,10\right)\;\text{with}\;0\leq \epsilon_{\nu }\leq \infty$$

where 𝒩( *μ*,*σ*) is a normal distribution with mean *μ* and dispersion *σ* and *x*_*i*,*m*_ ∈ {−3, −1.5} is the epileptogenicity prior for each brain region. Some prior probabilities are truncated by setting a possible minimum value. The likelihood function is given by

$$P\left(\nu \left[t\right]∼x,\theta \right)∼𝒩\left(\tilde{\nu },\epsilon_{\nu }\right)$$

where *ν* and $\tilde{\nu }$ are the empirical and estimated SEEG data features. Both the seizures envelopes and the simulated SEEG channel power were considered to be the data feature here. Two algorithmic diagnostic metrics are (i) goodness of convergence, the number of runs that terminate properly (the varying of the likelihood converges to the given threshold), and (ii) goodness of fit equals to 1 − $\frac{\sum \left(\text{var}\left(\nu -\tilde{\nu }\right)\right)}{\sum \left(\text{var}\left(\nu \right)\right)}$, where the sum is across all the SEEG recorded channels.

### Calculation of Epileptogenic Values

Brain region-specific epileptogenicity values (EVs) are computed based on estimated source time series resulting from the optimization pipeline, just like Wang et al. (2023). The onset of the seizure *t*_*i*_ in the region *i* corresponds to the first occurence of its source time series (variable *x* of the 2D Epileptor) of region *i* values above a threshold, set at 0 for empirical data. We set *t*_0_ = *min*(*t*_*i*_), *i* = 1, …, 162. When there are no values above 0, meaning no estimated seizure in a brain region, the onset value is set to *t*_*i*_ = 200. We calculate the *EV*_*i*_ of brain region *i* by

$${EV}_{i}=-\log\left(\frac{\left(t_{i}-t_{0}\right)+1}{20}\right).$$

Once the EV vector is normalized to [0, 1] for each optimization run, the optimization pipeline gives the distribution of EVs while considering the sensitivity of the sensor spatial sampling.

### Sensor Sensitivity

Measure of estimated EVs confidence was obtained from bootstrapping in the optimization pipeline. The measure evaluates the robustness of the identified source with regard to sensor location sensitivity. The bootstrapping approach relies on a leave-one-out approach, removing one randomly chosen sensor in each bootstrapping sample, while the optimization algorithm is run on the remaining data. The procedure is repeated 100 times using a random SEEG contact selection process with replacement, exactly in the same manner that Wang et al. (2023). All EVs are normalized by subtracting the minimum median EV and dividing by the difference between the maximum and minimum median EVs.

### Statistical Analysis

#### Performance scores.

Usually, the performance scores used to evaluate binary classification models, especially when the dataset is imbalanced are derived from the confusion matrix. The confusion matrix summarizes the correct and the wrong predictions and thus helps to understand the number of predictions made by a model for each class, and the classes to which those predictions actually belong. It helps to understand the kind of prediction errors the model made. Figure 3 illustrates this matrix, the derived rates, and formula of performance scores used here: true positive rate, false positive rate, true negative rate, and false negative rate. Those rates are then used to compute precision (percentage of correct prediction of the positive class) and recall (percentage of correct predictions for the positive class out of all positive predictions).

Balanced accuracy, the imbalanced data adapted accuracy, is the arithmetic mean of sensitivity and specificity (Kelleher, Namee, & D’Arcy, 2015). The classic accuracy score tends to be inflated due to the imbalanced nature of the dataset, which balanced accuracy prevents. The F-beta score used in this project is the harmonic mean of recall and precision (Baeza-Yates & Ribeiro-Neto, 1999) (Supporting Information Figure S8). As we are more interested in the precision than in recall, we gave more importance to precision than to recall, setting beta = 0.5. These two metrics were first used in combination during parameter tuning procedure as scoring functions, as well as to evaluate VEP pipeline EZN estimation. The model selection is based on balanced accuracy.

#### VEP outcome analysis.

Over the nine patients of the test dataset that we virtualized using all the described priors (VEP-EI, VEP-M, VEP-W, Na-MRI-prior 1, Na-MRI-prior 2, VEP-no-prior), the VEP pipeline provides results for a total of 26 seizures. In order to evaluate the performance of each prior, we compute the balanced accuracy and the *F*_0.5_-score for each seizure, using VEP-EI results as reference. Next, we compared the resulting balanced accuracy and *F*_0.5_-score with a bootstrapped (1,000,000 resampling) paired *t* test comparing each pair of priors. We used the classical threshold of 0.05 for the *p* value, also considering the threshold of 0.01.

## ACKNOWLEDGMENTS

The authors would like to thank L. Pini, C. Costes, and V. Gimenez for data acquisition and study logistics. We would also like to thank M. Woodman, V. Sip, A. Vattikonda, and M. Hashemi for helpful discussions.

## SUPPORTING INFORMATION

Supporting information for this article is available at https://doi.org/10.1162/netn_a_00371. The data that support the findings of this study are available on request from the corresponding author. The data are not publicly available due to sensitive information that could compromise the privacy of research participants.

## AUTHOR CONTRIBUTIONS

Mikhael Azilinon: Conceptualization; Data curation; Formal analysis; Investigation; Methodology; Visualization; Writing – original draft; Writing – review & editing. Huifang E. Wang: Data curation; Formal analysis; Funding acquisition; Investigation; Methodology; Software; Supervision; Writing – original draft; Writing – review & editing. Julia Makhalova: Data curation; Formal analysis. Wafaa Zaaraoui: Resources. Jean-Philippe Ranjeva: Validation; Writing – review & editing. Fabrice Bartolomei: Funding acquisition; Resources; Writing – review & editing. Maxime Guye: Conceptualization; Funding acquisition; Supervision; Writing – review & editing. Viktor Jirsa: Conceptualization; Funding acquisition; Project administration; Supervision; Validation; Writing – original draft; Writing – review & editing.

## FUNDING INFORMATION

Maxime Guye, 7TEAMS Chair. Mikhael Azilinon, Aix–Marseille University – A*MIDEX, Award ID: AMX-19-IET-004. Mikhael Azilinon, ANR, Award ID: ANR-17-EURE-0029. Fabrice Bartolomei, EPINOV, Award ID: ANR-17-RHUS-0004. Viktor Jirsa, Horizon 2020 Framework Program for Research 601 and Innovation, Award ID: 945539. Viktor Jirsa, Horizon 2020 Framework Program for Research and Innovation, Award ID: 785907. Viktor Jirsa, Horizon 2020 Framework Programme (https://dx.doi.org/10.13039/100010661), Award ID: No. 101147319. Huifang E. Wang, Aix–Marseille University – A*MIDEX, Award ID: AMX-22-RE-AB-135.

## Supplementary Material



## TECHNICAL TERMS

Virtual epileptic patient (VEP): Module simulating epileptic patient’s brain activity to assist clinicians in the identification of the epileptogenic zones (EZN).
Neural mass models (NMM): A straightforward approach to modeling the activity of neuronal populations, the so-called neural masses, described as groups of neurons with a common function and with similar or equal in-going and out-going connections.
Epileptor: Phenomenological model based on a system of coupled nonlinear differential equations generating epileptic dynamics.
VEP priors: Priors providing the location of the initiation of the seizures based on SEEG recordings processing.
^23^Na-MRI: Imaging technique allowing to quantify human brain sodium in vivo.
Maximum a posteriori probability (MAP): Estimate and unknown quantity corresponding to the mode of the posterior distribution.
Na-MRI priors: Priors providing the location of the initiation of the seizures-based machine learning model (beforehand trained with ^23^Na-MRI) predictions.
Spectral embedding: A nonlinear dimensionality reduction using Laplacian eigenmaps, which preserves the local geometry.
Nested cross-validation: Also called double cross-validation, this procedure uses two k-fold type loops, meaning that each training fold is also folded.
