# Supplementary material for: Brain sodium MRI-derived priors support the estimation of epileptogenic zones using personalized model-based methods in epilepsy
Source: Netw Neurosci. 2024 Oct 1;8(3):673–96. doi: 10.1162/netn_a_00371 (PMC11340996; doi:10.1162/netn_a_00371)
Supplement: Supplementary file 1 [file netn-8-3-673-s001.pdf]

Supplementary material

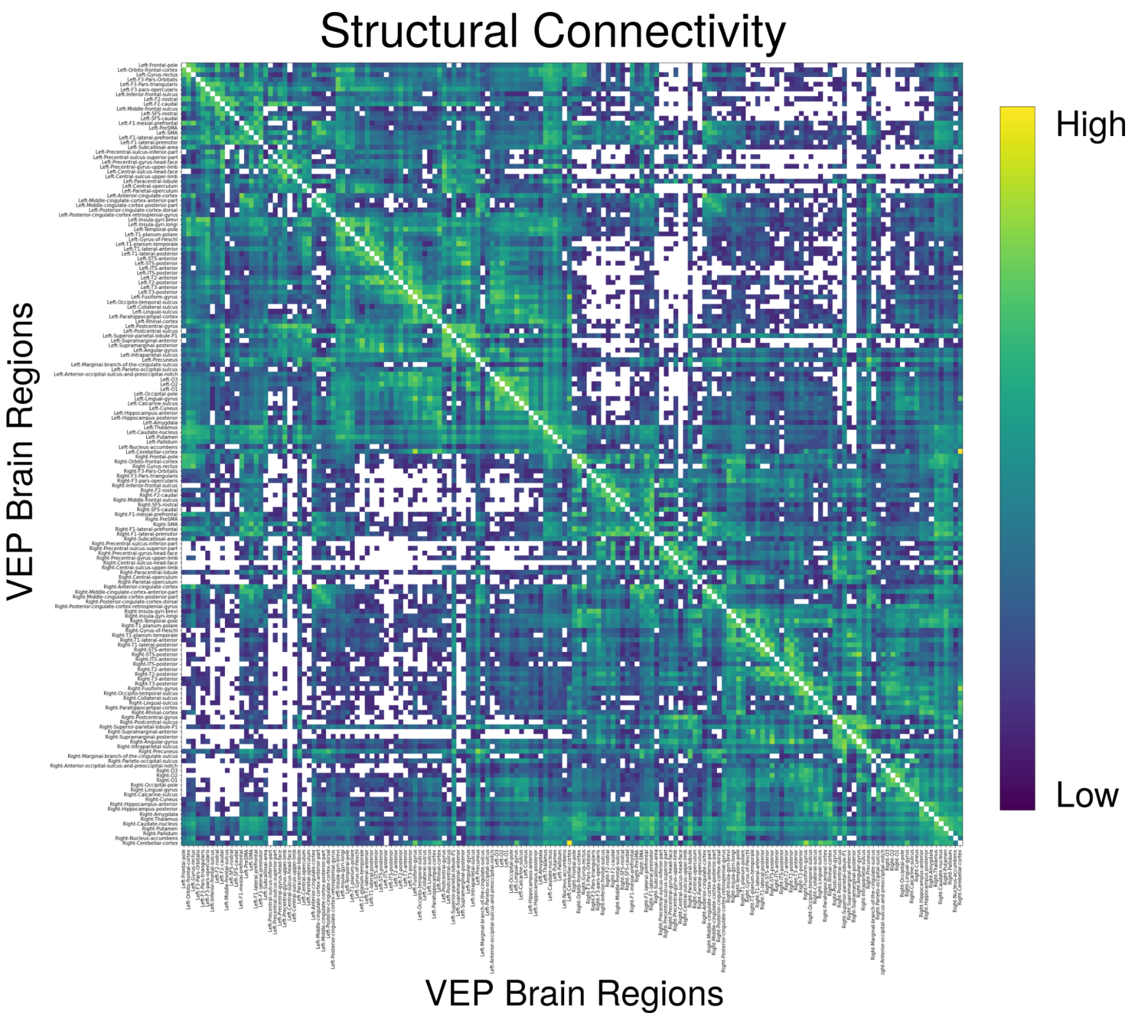

Figure 1: Structural connectivity for the clinical case between 162 VEP brain regions.

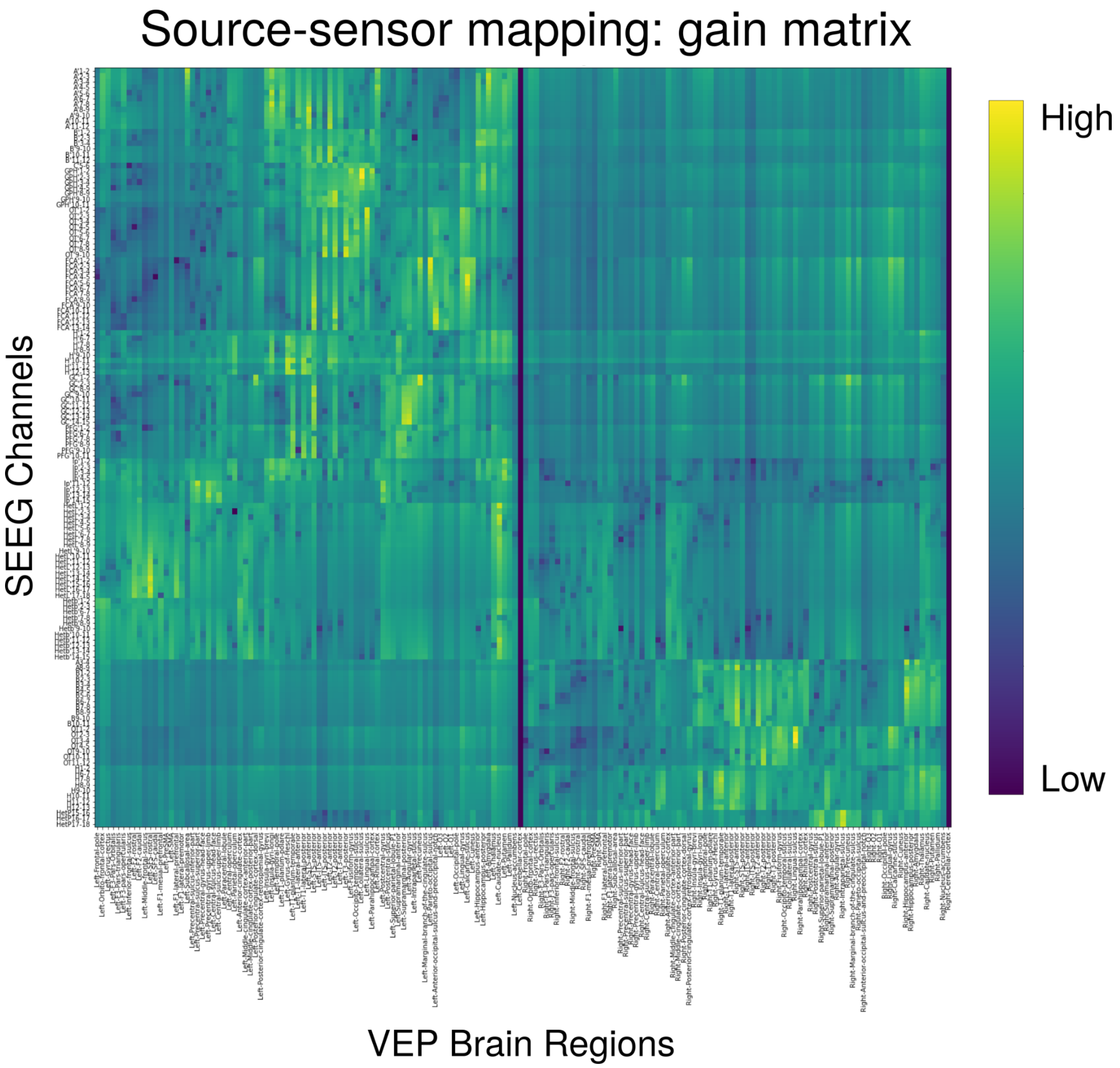

Figure 2: Source-to-sensor mapping (gain matrix) for the clinical case between recordings from SEEG bipolar electrodes and 162 VEP brain regions.

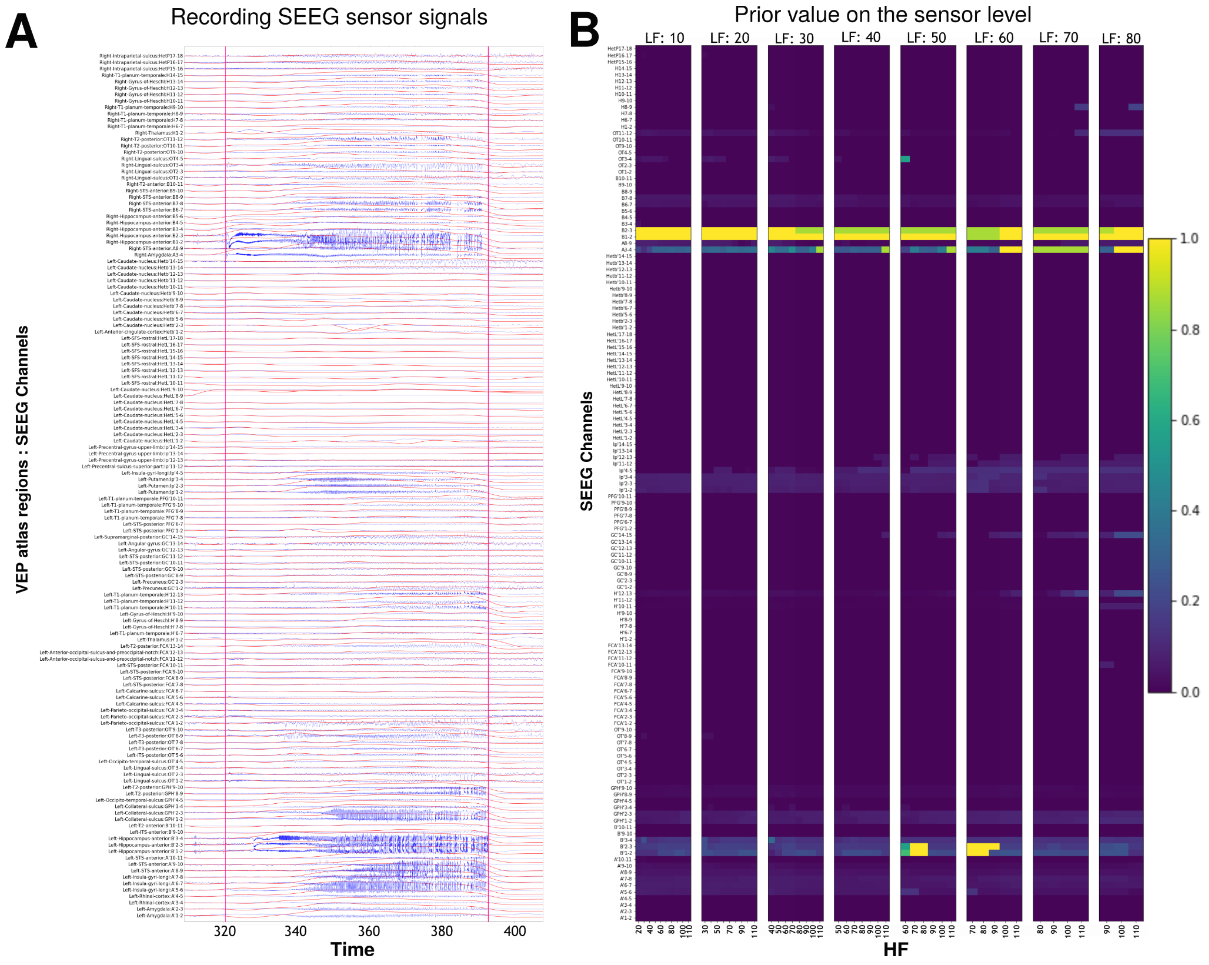

Figure 3: (A) Raw time series of the SEEG sensor signals from the example patient (in blue) and the corresponding data feature (in red). (B) The prior values of the sensors on 52 frequency bands; the low frequency boundary is from 10 Hz to 80 Hz with steps of 10 Hz, and the high frequency boundary is from 20 Hz to 110 Hz with steps of 10 Hz.

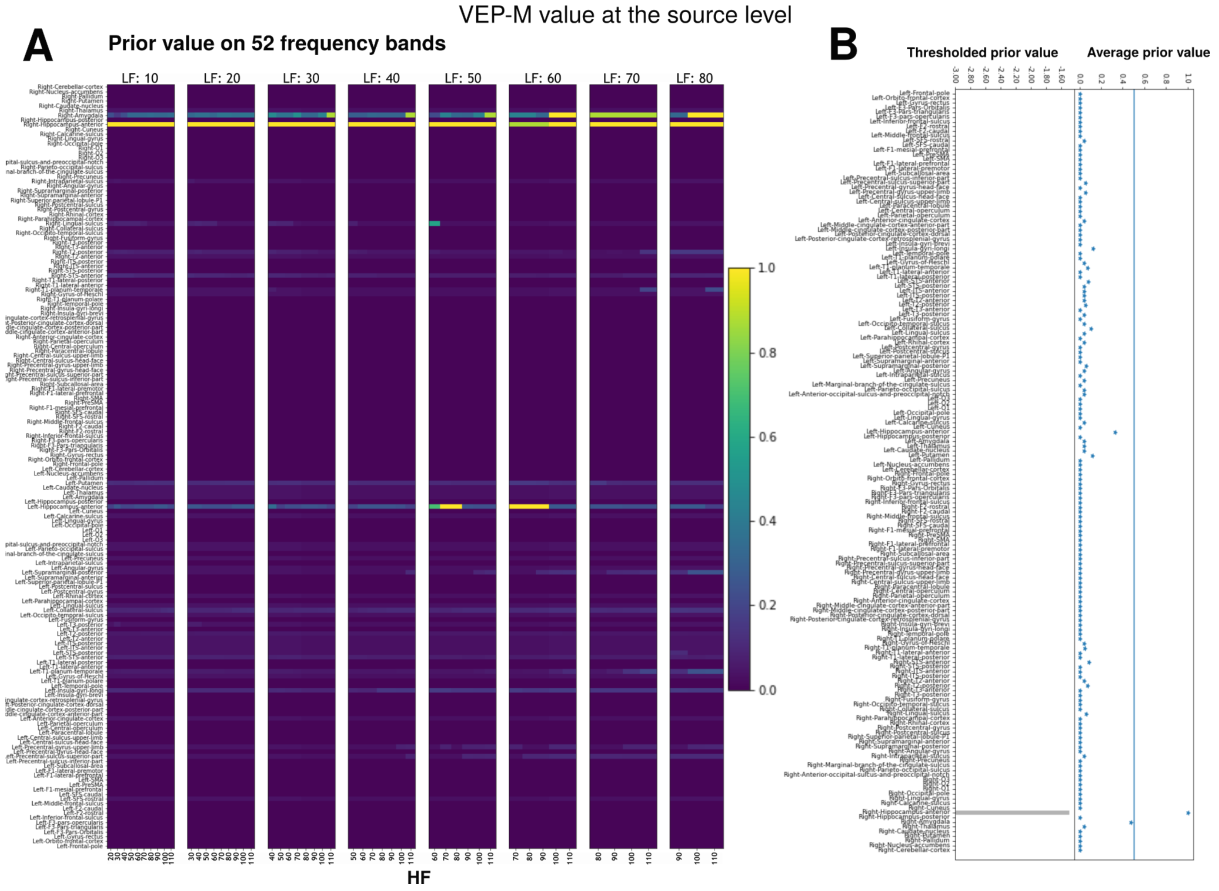

Figure 4: Prior value on the source level through the VEP-M algorithm. (A) For each frequency band (as previously defined in Figure 5.B.) sensor prior value is mapped to the closest source (i.e. that has the minimal distance). (B) The bars of the prior values on the left represent the prior values used in the model inversion module for the VEP-M run. The threshold was 0.5. On the right, the stars represent the average across 52 frequency bands of prior values on brain regions.

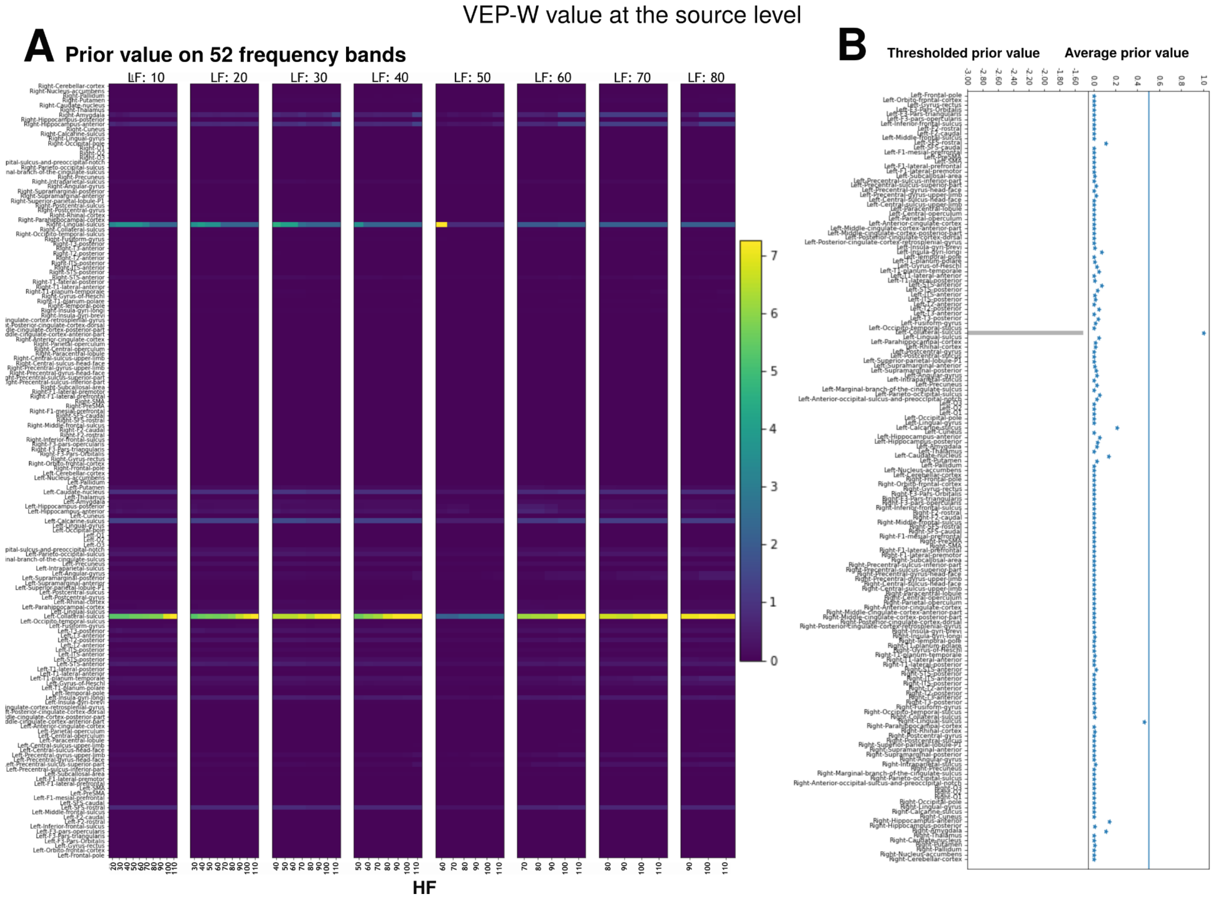

Figure 5: Prior value on the source level through the VEP-W algorithm. (A) For each frequency band (as previously defined in Figure 5.B.) weighted sum of all sensors prior value is mapped to each source (i.e. that has the minimal distance). The weight is based on the distance between the source and the sensors. (B) The bars of the prior values on the left represent the prior values used in the model inversion module for the VEP-W run. The threshold was 0.5. On the right, the stars represent the average across 52 frequency bands of prior values on brain regions.

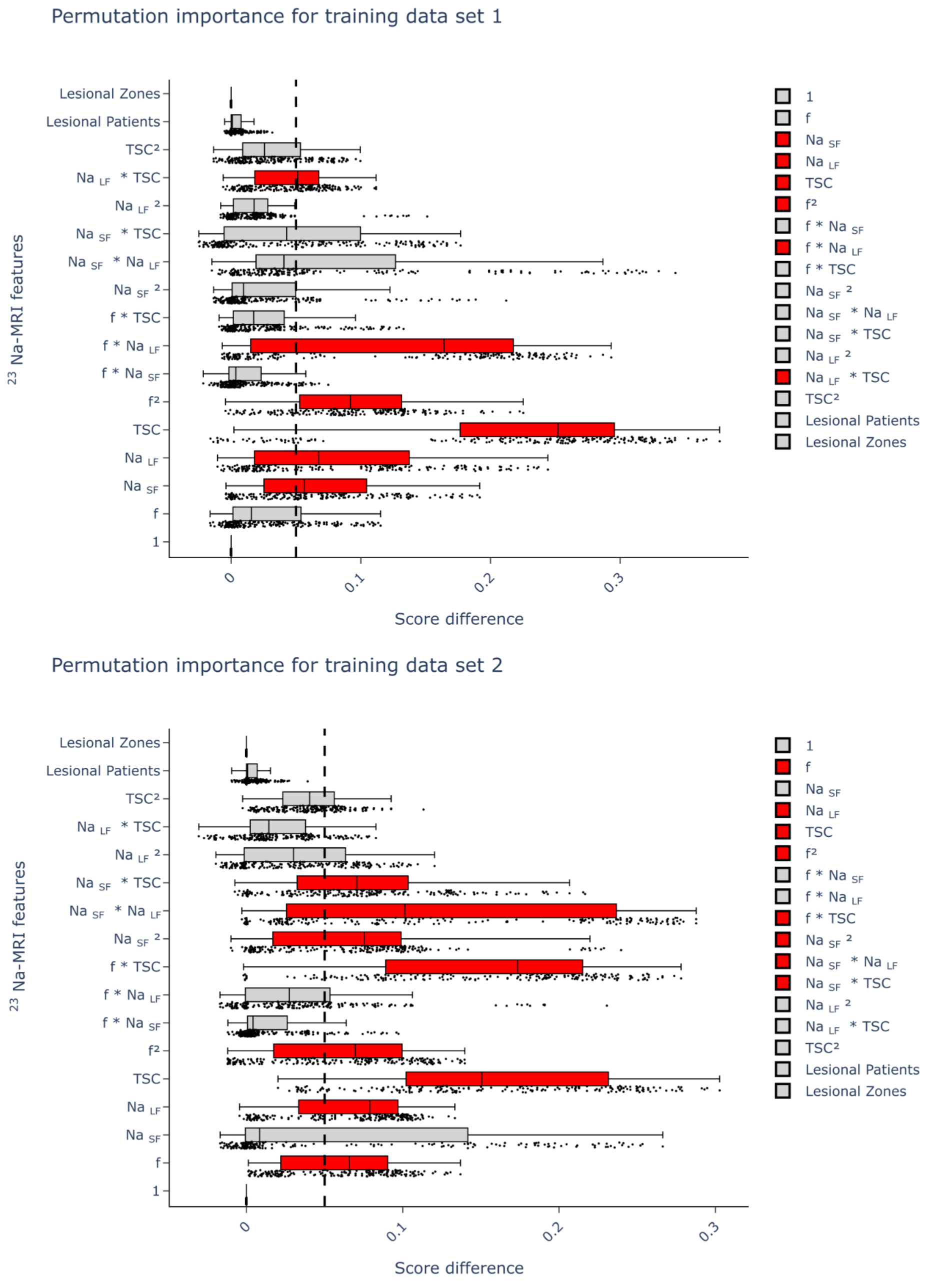

Figure 6: Permutation Feature Importance performed on ^{23}Na-MRI features for training dataset 1 (top) and 2 (bottom). Dot-line represents the threshold of 0.5 of score difference, used to select the features to be used in the models. The selected features are highlighted in red.

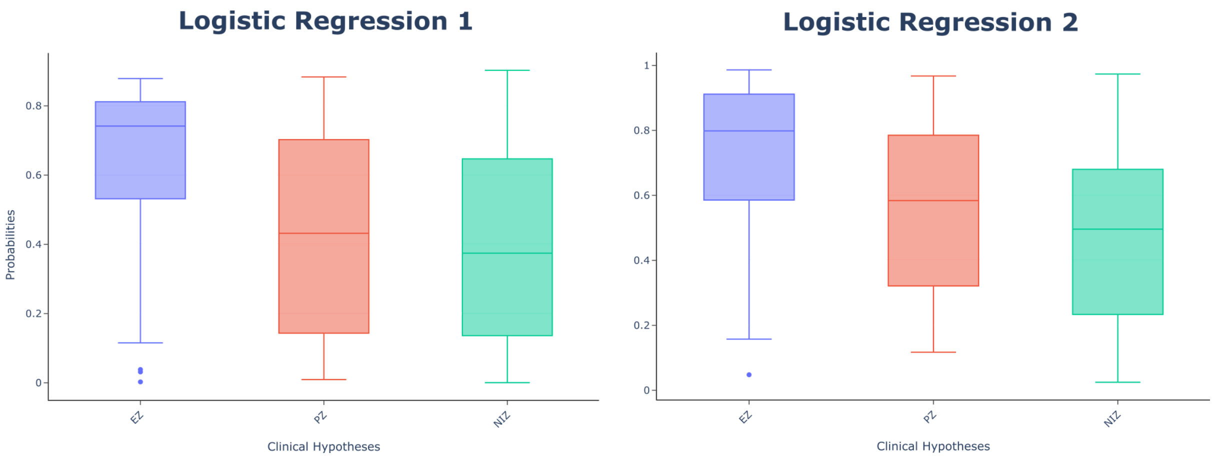

Figure 7: Logistic regression 1 (left) and 2 (right) prediction probabilities per brain region. Brain regions are categorized based on clinical hypotheses, giving 3 categories: epileptogenic zones (EZ) in blue, propagation zones (PZ) in red, and non-involved zones (NIZ) in green.

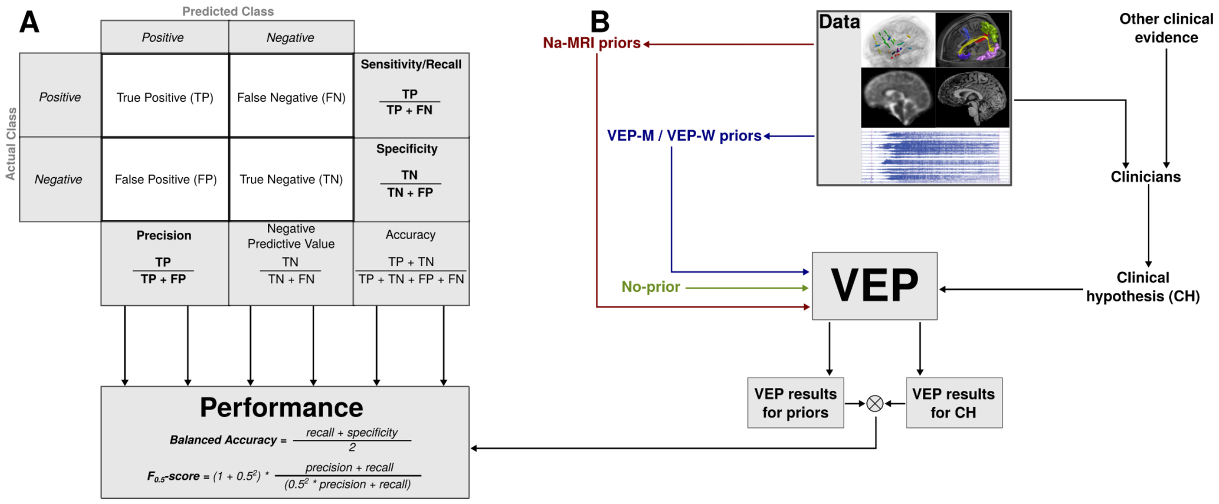

Figure 8: Performance evaluation process. The confusion matrix (A) is used to compute Balanced accuracy and $F_{0.5}$-score, the two performance evaluation metrics we explored. Data-driven VEP simulation through the priors (B) provided simulations from the priors and simulation from the CH, confronted altogether to evaluate the performance.

Table1: Patients clinical informations

Patient	Gender	Age	Age at epilepsy onset (y)	Epilepsy type	Side	Radiological diagnosis	Surgery made	Data Splits
1	F	24	10	Temporal latero-mesial	L	Normal	Yes	Training dataset 1
2	F	33	24	Temporal mesial	R	Normal	Yes	Training dataset 1
3	F	37	12	Temporal lateral	L	L superior temporal NDT	Yes	Training dataset 1
4	M	23	12	Temporal mesial	L	Normal	Yes	Training dataset 1
5	F	49	40	Temporal mesial	R	Normal	Yes	Training dataset 1
6	F	22	4	Bilateral temporal mesial, R insulo-operculo-frontal	R>L	Normal	No	Training dataset 1
7	M	36	29	Temporal mesial	R	Normal	Yes	Training dataset 1
8	F	30	8	Frontal	L	L lateral prefrontal FCD	Yes	Training dataset 1
9	F	56	30	Temporo-parietal mesial	R	R posterior cingulate FCD	No	Training dataset 2
10	M	44	13	Insulo-parietal	L	Normal	No	Training dataset 2
11	F	37	10	Temporo-insular	L	Normal	Yes	Training dataset 2
12	M	29	3	Temporal mesial	L	Normal	Yes	Training dataset 2
13	M	21	9	Temporo-insular	L	Normal	No	Training dataset 2
14	F	32	22	L parietal mesial & bilateral temporal mesial	R&L	L posterior cingulate NDT	Yes	Training dataset 2
15	F	27	17	Bilateral temporal mesial	R&L	Normal	No	Training dataset 2
16	M	30	19	Temporal Plus	L	Normal	No	Training dataset 2
17	F	29	0,1	Orbitofronto-insular	L	L orbitofrontal FCD	Yes	Testing dataset
18	M	15	11	Temporal latero-mesial	L	Normal	Yes	Testing dataset
19	M	17	7	Insulo-opercular	L	Normal	No	Testing dataset
20	M	42	12	Prefrontal Plus	R>L	Normal	No	Testing dataset
21	F	38	20	Temporal Plus	R	Periventricular nodular heterotopia	Yes	Testing dataset
22	M	52	40	Temporal Plus	R	Normal	Yes	Testing dataset
23	F	17	13	Temporal Plus	L>R	Bilateral periventricular nodular heterotopia	No	Testing dataset
24	M	30	22	Temporal Plus	R	Normal	Yes	Testing dataset
25	F	20	14	Insuli-opercular	R	Periventricular nodular heterotopia	No	Testing dataset
